# Effect of Home Visit Training Program on Growth and Development of Preterm Infants: A Double Blind Randomized Controlled Trial

**Published:** 2015-01

**Authors:** Mitra Edraki, Hossian Moravej, Masoume Rambod

**Affiliations:** 1 Community Based Psychiatric Care Research Center, Department of Pediatric Nursing, School of Nursing and Midwifery, Shiraz University of Medical Sciences, Shiraz, Iran;; 2 Department of Pediatrics, Nemazee Hospital, Shiraz University of Medical Sciences, Shiraz, Iran;; 3 Community Based Psychiatric Care Research Center, Department of Medical Surgical Nursing, School of Nursing and Midwifery, Shiraz University of Medical Sciences, Shiraz, Iran

**Keywords:** Development, Growth, Home visit, Preterm infant

## Abstract

**Background:** Home visit program can be effective in infants’ growth and development. The present study aimed to investigate the effect of home visit program on preterm infants’ growth and development within 6 months.

**Methods:** It was a double-blind clinical trial study. The study was conducted in Hafez, Hazrat-e-Zeinab, and Namazee Hospitals affiliated to Shiraz University of Medical Sciences, Shiraz, Iran from 2010 to 2011. Preterm infants were divided into intervention (n=30) and control groups (n=30) through blocked randomization. The intervention group received home visit training program for 6 months, while the control group only received the hospital’s routine care. Then, the infants’ growth indexes, including weight, height, and head circumference, and development criteria were compared on the first day of admission in Neonatal Intensive Care Unit, and then first, second, third, and sixth months. The data were analyzed using Chi-square, independent t-test, and repeated measures ANCOVA.

**Results:** The mean weight of the intervention and control group infants was 7207.3±1129.74 and 6366.7±922.26 gr in the sixth month. Besides, the intervention group infants’ mean weight was higher compared to the control group after six months (t=-3.05, P=0.03). Also, a significant difference was found between the two groups regarding development indexes, such as following moving objects with the head, keeping the head stable when changing the position from lying to sitting,  producing “Agha” sound, and taking objects by hand (P<0.05) during six months of age.

**Conclusion:** The results showed that the home visit program was effective in preterm infants’ weight gain and some development indexes at the sixth month. Considering the importance of infants’ growth and development, healthcare staff is recommended to incorporate home visit training into their programs, so that steps can be taken towards improvement of preterm infants’ health.

**Trial Registration Number:** IRCT2014082013690N3

## Introduction

Preterm birth and low birth weight increase the chance of early mortality.^1^ These newborns are faced with health problems and delay in development and also experience more cognitive, behavioral, psychological, and social problems compared to term newborns.^2^ They may also suffer from sensory, nervous, and motor deficiencies,^3^^,^^4^ which can in turn affect their growth and development in future.^5^ Evidence has indicated that preterm newborns had weaker cognitive, motor, and behavioral outcomes compared to term infants.^6^ Infant’s health predicts the adulthood health and other outcomes through life.^7^ Therefore, infantile and childhood disorders may accompany individuals up to adolescence and adulthood.^6^

The parents’ ability to adapt to a preterm infant and the quality of the relationship between the parents and the infant can affect the child’s growth and development in future.^8^ The primary relationship can also affect the parents’ emotions, understanding, and attitude toward the child’s future and needs.^8^ Home visit program is an interventional strategy which can involve a wide range of individuals, including parents and children^9^ and influence the parents’ emotions, understanding, attitude, and awareness. This strategy, as a supportive intervention, plays a critical role in diagnosis of social and physical problems as well as in beginning and continuation of breastfeeding during the first weeks after delivery.^10^ In addition, home visit during the first days after birth provides an opportunity for examining the mother and her infant, training health, infant’s nutritional support, provision of functional and emotional support, and reference of the individuals to other specialists if necessary.^11^^-^^13^ This program also improves maternal and infantile care performance, including beginning of breastfeeding, exclusive breastfeeding, skin to skin contact, paying attention to hygiene (washing hands and quality of water), taking care of the umbilical cord, and taking care of the infant’s skin.^14^ Furthermore, home visit is accompanied by reduction of need for re-hospitalization, improvement of mother’s health behaviors, earlier discharge, and decrease of treatment costs.^10^ Edraki et al. performed home visit program on preterm infants and reported that the number of hospitalized cases in the first six months of life was lower in the intervention group compared to the control group.^15^ Additionally, evidence has indicated that home visit program increases the length of exclusive breastfeeding.^16^ Moreover, the results of a meta-analysis demonstrated that home visit program improved the parents’ effective behaviors and children’s development outcomes.^17^

The American Academy of Pediatrics has recently supported home visit program in prenatal and postnatal periods and has considered this program as a method for improvement of children’s health and development.^18^^,^^19^ Researchers have also indicated this program as a strategy for mprovement of children’s health and development.^20^^,^^21^ Nonetheless, contradictory results have been obtained regarding the effect of this program on improvement of children’s health and development.^14^^,^^18^^,^^21^^,^^22^ Besides, a limited number of studies have been conducted on the effect of home visit program on health criteria, including growth and development, in preterm infants. Casey et al showed an early intervention on 8-year growth status of low-birth-weight preterm infants led to higher weights, heights, and head circumferences compared with control infants.^23^ A researcher demonstrated that parents’ training on the teenage mother had significant effects on weight and height, but not head circumference infants at 4 and 12 months. Moreover, they showed that parents’ training affects the infant’s temperament and cognitive development at 4, 8, and 12 months of age.^24^ On the other hand, another study indicated that four-month early intervention program in African American premature infants did not affect their weight, height, and head circumference.^25^ Since there are contradictory results about the early intervention on high risk infants and there were no study in Iran with the aim the effect of home visit program in the postnatal period on improvement of preterm infants’ growth and development, this study was conducted. The researchers hypothesized that the preterm infants that received home visit program during the first six months of life would present better growth and development compared to those receiving the routine care.

## Materials and Methods

*Design*

It was a double-blind and randomized controlled trial design study. The present experimental study with a control group aimed to determine the effect of home visit on preterm infants’ growth and development within the first six months of their life.

*Setting*

The study was conducted in Hafez, Hazrat-e-Zeinab, and Namazee hospitals affiliated to Shiraz University of Medical Sciences from April 2010 to May 2011.

*Participants*

The target population included all the preterm infants. The inclusion criteria of the study were mother’s age 18 years or above, gestational age<37 weeks, birth weight<2500 gr, being fed orally, living in Shiraz; also, during participation in this study the preterm infants and their parents should not be participating in another program or intervention, and their parents should not be one of the medical staff. On the other hand, the exclusion criteria of the study were suffering from brain disorders, congenital cardiovascular diseases, metabolic or endocrine disorders, and known genetic and chromosomal abnormalities and having previous history of preterm infants in their family. 

*Randomization and Sample Size*

Using a pilot study, based on power of 0.8, effect size=0.20, and α=0.05, a 60-subject sample size (30 subjects in each group) was determined for this study. In order to select the participants, all the preterm infants who had inclusion criteria were selected based on convenience sampling. Then, in order to allocate the subjects into the study groups, a blocked randomization procedure with a 2 block sizes were applied to provide a balance between the groups and prevent selection bias. Therefore, all the participants were randomly allocated into either intervention (n=30) or control group (n=30) through blocked randomization.

**Figure 1:**
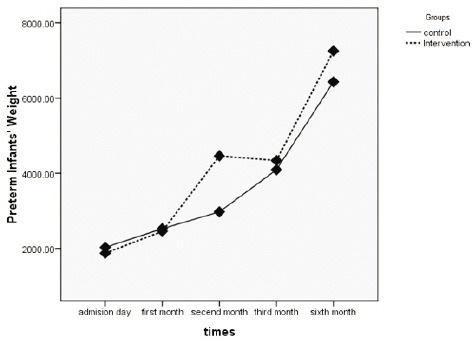
Flow diagrams of the preterm infants through six months of age of the study

During the study, in the control group, one preterm infant was excluded in the third month after birth and two subjects were withdrawn in the sixth month after birth. They were excluded due to death. However, all the subjects in the intervention group participated in the sixth month after birth intervention (Figure 1).

*Measures*

The data were collected using a data collection form including two sections. The first section involved demographic characteristics, including parents’ age at infant’s birth, infant’s gestational age, and infant’s sex, and mothers’ and fathers’ age and educational levels. The second part of the form included growth criteria, such as weight, height, head circumference, and development indexes, such as following moving objects with the head, social laughing, keeping the head stable when changing position from lying to sitting, producing “Agha” sound, taking objects by the hand, and laughing.

The infants’ weight was measured using a scale (Seca 354, Germany). In order for consistency in measurement, the scale was periodically calibrated before the study. In addition, the instrument was compared to a standard scale in order to increase its accuracy. Using inter-rater reliability, two individuals measured 10 infants’ weight using the above-mentioned scale independently, revealing a correlation coefficient of 0.90. Besides, the infants’ height and head circumference were measured using a tape meter made of fiber glass. Each centimeter of this tape meter included 10 lines. In order to increase the accuracy of this instrument, two individuals independently measured 10 infants’ height and head circumference, indicating the correlation coefficient to be 0.90.

*Outcome Measures*

The outcome measures of the study consisted of the demographic information and growth and developmental indexes. In order to assess growth, the infants’ weight, height, and head circumference were used. In addition, development indexes included following moving objects with the head, social laughing, keeping the head stable when changing position from lying to sitting, producing “Agha” sound, taking objects by the hand, and laughing. The parents were asked about these behaviors when they referred to Motahari educational clinic affiliated to Shiraz University of Medical Sciences at the first, second, third, and sixth months after birth. The data were collected on the first day of admission in Neonatal Intensive Care Unit (NICU), and then first, second, third, and sixth months after birth.

*The Intervention*

The preterm infants who met the inclusion criteria were enrolled into the study on admission to the ward or NICU. The mothers of both study groups were trained about the hospital’s standard care, similar to the mothers who had given birth to term infants. At discharge, the researchers got the parents’ living place address. 

*Home Visit Program for Intervention Group*

The home visit program was designed by a pediatrician, a pediatric nurse and a medical-surgical nurse. Then, the content validity of the program was approved by 5 faculty members of pediatrics department and pediatric nurses. The home visits intervention was provided by pediatric nurse (Master’s degree and faculty member).

The first home visit program which lasted for 20 minutes was performed on the first day after discharge and included familiarity with the differences between term and preterm infants, how to take care of a preterm infant, and how to take care of the umbilical cord. At the end of this session, the mothers were provided with an educational booklet about taking care of preterm infants, advantages of exclusive breastfeeding, nutrition, and bathing the infant. 

The second session was carried out the day after the first session and involved training about the advantages of breastfeeding, how to hold the infant, and nutrition for 20 minutes. After the training, the mother was required to breastfeed the infant in the presence of the interventionist. 

The third session was conducted 1 week after the first visit and included training about who to bath the infants and take care of them before and after taking bath. Then, the mother bathed the infant in the presence of the interventionist. 

Moreover, four visits were also provided at the first, second, third, and sixth months after birth. During these visits, training about taking care of the infant’s perineum, breastfeeding techniques, sufficiency or insufficiency of breast milk, problems related to mother’s breasts, how to take supplementary drugs, covering the infant, location for keeping the infant, bathing and cleaning the infant, and nutrition were done. Moreover, counseling and supportive care such as referring to neonatal clinic, neonatologist, nutritional and mental health specialist and were provided for parents.

*The Control Group*

In the control group, the information was obtained from the mothers at the first, second, third, and sixth months after birth. The control group mothers’ questions were answered, but no predefined program was performed in this group. It should also be noted that in case an infant had to be hospitalized, it was not excluded from the study.

*Blinding*

In this study, the researcher assistant who collected the data was masked to the study groups and the intervention program. Moreover, the statistician who did the data analysis was blinded to the allocation of the participants in the study groups, as well. 

*Ethical Considerations*

The present study was approved by the Ethics Committee of Shiraz University of Medical Sciences. At first, the study objectives were explained to the participants. Written informed consent was taken from the parents. The infants’ parents were also reminded that participation in the study was voluntary and were assured about the secrecy of their information. They were also ascertained that no risks threatened their infants during the study.

*Data Analysis*

The data were analyzed in SPSS statistical software (v. 16) using descriptive statistics, including number and percent, and inferential statistics, such as Chi-square, independent t-test, and repeated measures ANCOVA. In this study, the gestational age, and parental age and their educational status were considered as covariates. Moreover, P<0.05 was considered as statistically significant. 

## Results

The mean age of the mothers was 27.17±5.14 years in the intervention group and 25.31±6.19 years in the control group. The fathers’ mean age in the intervention and control groups were 31.34±5.40 and 29.79±4.32, respectively. Half of the participants in the intervention group and %53.3 of the control group were males. In addition, the infants’ birth weight was 1860.0±560.35 and 2084.0±298.45 gr in the intervention and the control group, respectively. Most of the mothers’ and fathers’ educational levels were diploma and college degree. The results of this study showed no significant differences between the two groups regarding mothers’ and fathers’ age, and educational levels. Moreover, the two groups were also similar with respect to gestational age and infants’ birth weight and gender (Table 1).

**Table 1:** Demographic characteristics of the preterm infants and parents in the intervention and control groups

**Variables**	**Intervention**	**Control**	**test, P value**
Infants’ birth weight			
Mean±SD	1860.0±560.35	2084.0±298.45	t=1.93, P=0.06
Gestational age			
Mean±SD	35.10±2.79	35.60±1.61	t=0.84, P=0.39
Mothers’ age			
Mean±SD	27.17±5.14	25.31±6.19	t=0.98, P=0.32
Fathers’ age			
Mean±SD	31.34±5.40	29.79±4.32	t=-1.20, P=0.23
Mother’s education, n (%)			
High school and lower	14(48.3)	11(37.9)	χ^ 2 ^=6.46,P=0.16
Diploma and College degree	15(51.7)	18(62.1)	
Father’ education, n (%)			
High school and lower	12(41.4)	11(37.9)	χ^ 2 ^=4.33, P=0.36
Diploma and College degree	17(58.6)	18(62.1)	
Infant’s sex, n (%)			
Male	14(46.7)	15(50.0)	χ^ 2 ^=0.06, P=0.79
Female	16(53.3)	15(50.0)	

**Figure 2:**
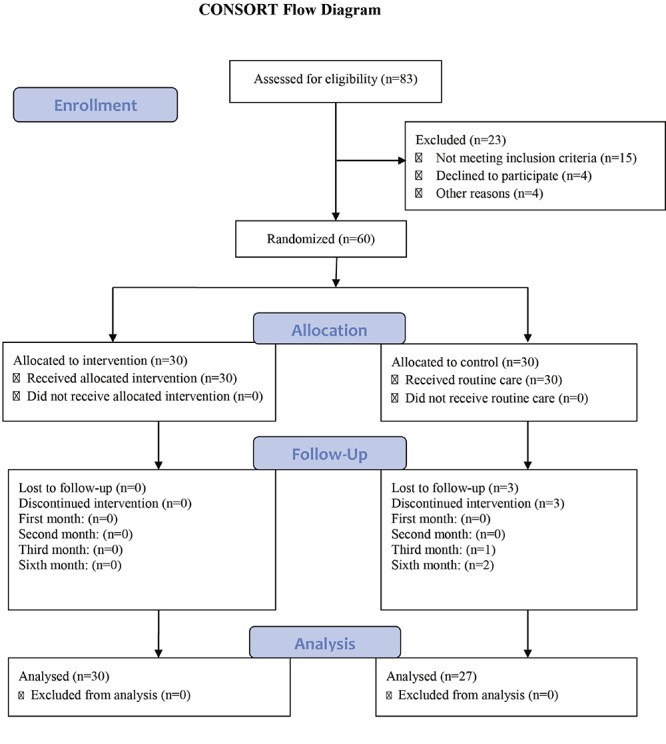
Preterm infants’ weight repeated measures ANCOVA between the intervention and control groups across the fifth study periods

The results of independent t-test also showed no significant difference between the two groups concerning the infants’ mean weight at one, two, and three months of age. Nonetheless, a significant difference was observed between the two groups after six months (t=-3.05, P=0.03); and the intervention group infants showed higher weight gain compared to the control group. However, the results of repeated measures ANCOVA indicated no significant difference between the two groups’ weight in the five measurements (Table 2 and Figure 2). On the other hand, the findings showed a significant difference within the groups’ weight in the five measurements. The within subject test indicated that there was a significant time effect, in other words, the groups changed in infants’ weight over time (Table 2).

**Table 2:** Growth indexes (weight, height, and head circumference) of the preterm infants in the two groups at admission day in NICU, and one, two, three, and six months of age

**Growth indexes**	**Admission day in NICU**	**First month**	**Second month**	**Third month**	**Sixth month**	**Repeated measures ANCOVA†**	
	**Mean±SD**	**Mean±SD**	**Mean±SD**	**Mean±SD**	**Mean±SD**		
						**Between groups**	**Within groups**
Weight						F=2.24 P=0.14	F=16.32 P<0.0001
Intervention group	1860.0± 560.35	2412.3± 724.71	4178.3± 581.3	4323.7± 863.84	7207.3± 1129.74		
Control group	2050.7± 221.10	2498.3± 530.04	3131.0± 635.50	3976.9± 822.09	6366.7± 922.26		
Between group analysis, P	t=1.73, P=0.08	t=0.52, P=0.60	t=-0.98, P=0.33	t=-1.57, P=0.12	t=-3.05, P=0.03^*^		
Head circumference						F=0.16 P=0.86	F=0.58 P=0.44
Intervention group	30.03± 1.92	32.31± 2.23	33.84± 2.30	35.75± 2.38	40.37± 3.45		
Control group	30.43± 1.67	32.83± 2.03	34.60± 2.43	36.46± 2.75	40.17± 3.18		
Between group analysis, P	t=0.86, P=0.39	t=0.93, P=0.35	t=0.98, P=0.33	t=0.74, P=0.46	t=-0.22, P=0.82		
Height						F=1.56 P=0.21	F=0.002 P=0.98
Intervention group	41.63± 7.22	44.69± 7.69	47.31± 7.91	51.23± 8.07	60.02± 7.45		
Control group	44.30± 5.42	46.23± 3.27	49.70± 4.45	53.55± 4.78	62.27± 6.34		
Between group analysis, P	t=1.61, P=0.11	t=0.98, P=0.32	t=1.43, P=0.15	t=1.33, P=0.18	t=1.20, P=0.23		

*Significant, †Gestational age, and parental’ age and educational status were considered as covariates.

In this study, no significant difference was observed between the two groups concerning the infants’ mean height on the first day of admission in NICU. Moreover, the mean of infants’ height was not different between the intervention (44.69, SD=7.69) and the control (46.23, SD=3.27) groups in the first month of age. In addition, the mean of infants’ height was not significantly different between the two groups in the second, third, sixth month of infants’ age (P>0.05). Moreover, no difference was found between and within the two groups in the fifth measurements (F=1.56, P=0.21) (Table 2).

The mean of the infants’ head circumference was 30.03 (SD=1.92) in the intervention and 30.43 (SD=1.68) in the control groups on the first day of admission. The differences between the two groups on the first day of admission were not significant (F=0.86, P=0.39) (Table 2). The changes of infants’ head circumference across the five measurements had the same patterns in both groups. The results of repeated measures ANCOVA did not show a significant difference between and within the two groups regarding the infants’ mean scores of head circumference during the five measurements (F=0.16, P=0.86) (Table 2).

As to the development indexes, the study results showed a significant difference between the two groups regarding following moving objects with the head, keeping the head stable when changing position from lying to sitting, producing “Agha” sound, and taking objects by the hand so that these behaviors developed earlier in the intervention group compared to the control group (P<0.05) during six months of age. Nonetheless, no significant difference was found between the two groups with regards to social laughter and laughing (P>0.05) during six months of age (Table 3).

**Table 3:** Development indexes of the preterm infants in the two groups during six months of age

**Development indexes**	**Intervention group**	**Control group**	**t-test,** **P-value**
	**Mean±SD**	**Mean±SD**	
Following moving objects with the head	1.36±0.49	2.75±0.87	t=7.58, P<0.001*
Social laughs	3.43±1.35	3.51±1.52	t=0.22, P=0.82
Keeping the head stable when changing position from lying to sitting	2.40±0.93	2.79±4.90	t=2.01, P=0.04*
Making “Agha” sound	3.86±1.41	5.03±1.48	t=2.84, P=0.006*
Taking objects by the hand	3.80±1.49	5.55±1.08	t=5.02, P<0.001*
Laughing	5.13±1.43	5.59±1.11	t=1.33, P=0.18

*Significant

## Discussion

The present study aimed to investigate the effect of home visit in the first six months of birth on preterm infants’ growth and development. The study results revealed a significant difference between the two groups regarding the weight at six months of age. The results also indicated that following moving objects with the head, keeping the head stable when changing position from lying to sitting, producing “Agha” sound, and taking objects were developed earlier in the intervention group compared to the control group during six months of age.

The results of this study demonstrated that the intervention group infants’ mean weight was significantly higher compared to the control group in sixth month of age. In the same line, Foroud and Foroud conducted a study on the effect of home visit on infants and indicated that home visit in the first six months of birth led to more weight gain compared to the control group.^26^ Consistently, Pabarja et al. reported that the mean weight gain was higher in the home visit group in comparison to the control group between the first and second months after birth.^27^

The findings of the current study showed that home visit resulted in faster development of behaviors, such as following moving objects with the head, keeping the head stable when changing position from lying to sitting, producing “Agha” sound, and taking objects, in the first six months of life. Similarly, the results of the study by Caldera et al. in 2007 revealed that the children receiving home visit during the first two years of life showed better behavioral and developmental outcomes compared to the control group.^9^ In addition, Lowell et al. carried out a research in 2011 and reported that home visit program was effective in the infants’ language development.^28^^,^^29^ Moreover, another study revealed that initial interventions since discharge up to three years of age increased the infants’ cognitive development scores in comparison to the control group at the ages of 2 and 3 years. Slight changes were also observed in their behavioral outcomes. However, the results showed no significant difference between the two groups with respect to behavioral and cognitive development at 5 and 8 years of age.^30^^,^^31^ The findings of a meta-analysis also demonstrated that early intervention programs were effective in improvement of infants’ cognitive outcomes, but they were less influential on motor outcomes and school age.^32^ Hence, home visit program seems to affect achieving family needs, leading to positive changes for both parents and children.

The present study had some limitations, the first of which being the small sample size. Thus future studies with larger sample size are recommended. Considering the reduction in the number of preterm infants in the recent years and due to the parents’ unwillingness to let the researcher enter their houses, the researchers were faced with difficulties in collection of the data. Another limitation of the study was related to the inclusion criterion of “living in Shiraz”, which might affect the generalizability of the results. Thus, further studies are recommended to be conducted in other urban and rural regions in different parts of the country. The last study limitation was its short follow-up period. With respect to the importance of this follow-up and its probable effects on children’s health, such infants are recommended to be followed up until school age and even adolescence. 

The outcomes of this study were measured by developmental indexes, since there are some reliable and valid instruments such as Bayley Scales for of Infant and Toddler Development and Dr. T. Berry Brazelton Scale (The Neonatal Behavioral Assessment Scale), other studies to assess developmental indexes by these instruments are recommended.

The present study investigated the impact of home visit after infants’ birth. Thus, future studies are suggested to assess the effect of home visit educational program during pregnancy on children’s development, beginning of breastfeeding, continuation of breastfeeding, and length of breastfeeding. Moreover, the current study evaluated the effect of home visit on preterm infants. Therefore, another study is required to assess the effect of home visit on term infants and compare it to that of preterm ones.

## Conclusion

The present study showed that home visit training program increased preterm infants’ weight gain in six months after birth and improved many developmental indexes. Therefore, this program is recommended to be considered for preterm infants, so by improving growth and development in these infants, health promotion occurs. More studies with evidence based practice in this regard are suggested. 
